# A Short Progress Report on High-Efficiency Perovskite Solar Cells

**DOI:** 10.1186/s11671-017-2187-5

**Published:** 2017-06-14

**Authors:** He Tang, Shengsheng He, Chuangwei Peng

**Affiliations:** 1 State Key Laboratory of Electronic Thin Films and Integrated Devices, University of Electronic Science and Technology of China, Chengdu, 610054 China; 2 School of Microelectronics and Solid-State Electronics,University of Electronic Science and Technology of China, Chengdu, 610054 China

**Keywords:** Solar cells, Perovskites, Stability, Renewable energy

## Abstract

Faced with the increasingly serious energy and environmental crisis in the world nowadays, the development of renewable energy has attracted increasingly more attention of all countries. Solar energy as an abundant and cheap energy is one of the most promising renewable energy sources. While high-performance solar cells have been well developed in the last couple of decades, the high module cost largely hinders wide deployment of photovoltaic devices. In the last 10 years, this urgent demand for cost-effective solar cells greatly facilitates the research of solar cells. This paper reviews the recent development of cost-effective and high-efficient solar cell technologies. This report paper covers low-cost and high-efficiency perovskite solar cells. The development and the state-of-the-art results of perovskite solar cell technologies are also introduced.

## Introduction

About 85% of the world’s energy requirements are currently satisfied by exhaustible fossil fuels that have detrimental consequences on human health and the environment. Moreover, the global energy demand is predicted to double by 2050 [1].

**Fig. 1:**
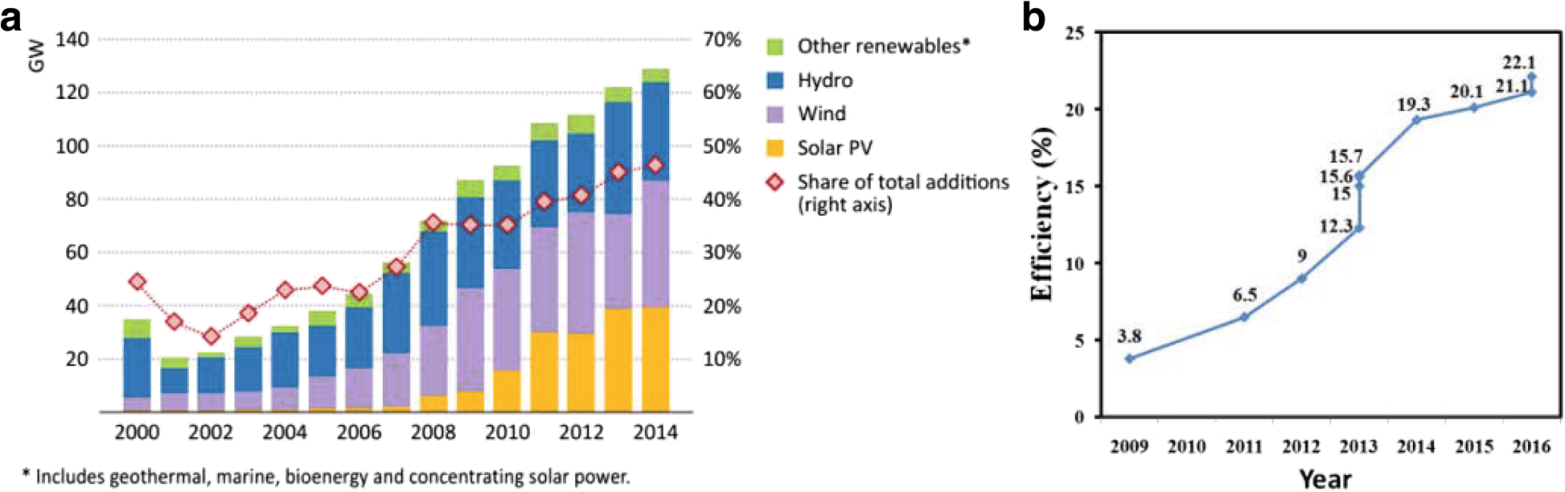
**a** Global renewable-based power capacity additions by type and share of total capacity additions [60]. **b** Rapid PCE evolution of perovskite solar cells from 2009 to 2016

Therefore, the development of renewable energy, such as wind energy, water energy, and solar energy, becomes an imminent requirement. Renewable energy-based power generation capacity is estimated to be 128 GW in 2014, of which 37% is wind power, almost one third solar power, and more than a quarter from hydropower (Fig. 1 a). This amounted to more than 45% of world power generation capacity additions in 2014, consistent with the general upward trend in recent years.

Due to abundance, low cost, and environmental friendliness, solar energy attracts increasingly more attention from all over the world, which makes the rapid development of solar cell research in recent years.

In general, a commonly used classification divides the various PV technologies (in commercial as well as in R&D stage) into three generations [2]: first generation, G1: wafer-based; mainly mono c-Si and mc-Si ; second generation, G2: thin film; a-Si, CdTe, CIGS, CuGaSe; third generation, G3: multi-junction and organic photovoltaics (OPV), dye-sensitized solar cells (DSSCs), and solar cells based on quantum dots as well as other nano-materials.

The development of the three-generation solar cells produced a rich variety of solar cells, such as Si solar cells, III–V solar cells, perovskite solar cells (PSCs), thin film solar cells, dye-sensitized solar cells, and organic solar cells. However, practical, low-cost, and high-efficiency third-generation solar cells are yet to be demonstrated. Si solar cells are well developed and mature, but there is little room for further improvement [3–6]. III–V solar cells have a very high efficiency; however, its weakness is the high cost, which limits its applications [7–9]. Quantum dot solar cells have been receiving significant attention because of their low cost and high efficiency, but most efficient devices have been prepared with toxic heavy metals of Cd or Pb [10–12]. Halide perovskites have recently emerged as promising materials for low-cost, high-efficiency solar cells. As the perovskite solar cell technology becomes more and more mature, the efficiency of perovskite-based solar cells has increased rapidly, from 3.8% in 2009 to 22.1% in 2016 [13–16]. However, the stability issues still require further studies.

To give an update of the field, this paper reviews the recent development of high-efficiency PSCs. This report briefly introduces the history of PSCs and then focuses on the key progress made in high-efficiency perovskite solar cells. Recent efforts on the stability of perovskite solar cells will also be discussed. At the end of the report,we also give a brief introduction to the interface engineering.

## Principle and History of Perovskite SCs

PSCs have recently become one of the hot spots owing to its low preparation cost and high-conversion efficiency in the fields of solar cell research. And it is regarded as a great potential material for its superiority (compared with other materials) that may assist perovskite with ultimate usurping of the reigning cell material.

In 1991, inspired by the principle of photosynthesis, O’Regan and Gratzel reported a landmark construction of solar cell called dye-sensitized solar cell, which can cover the sun light energy into electricity energy with an efficiency about 7% [17]. Presenting numerous advantages such as abundant raw materials, facile processing, and low cost compared with conventional solar cells, these novel solar cells made itself investigated popularly rapidly after its arising. And it is this work that inspired the emergence of PSCs, a DSSC with perovskite compounds.

**Fig. 2:**
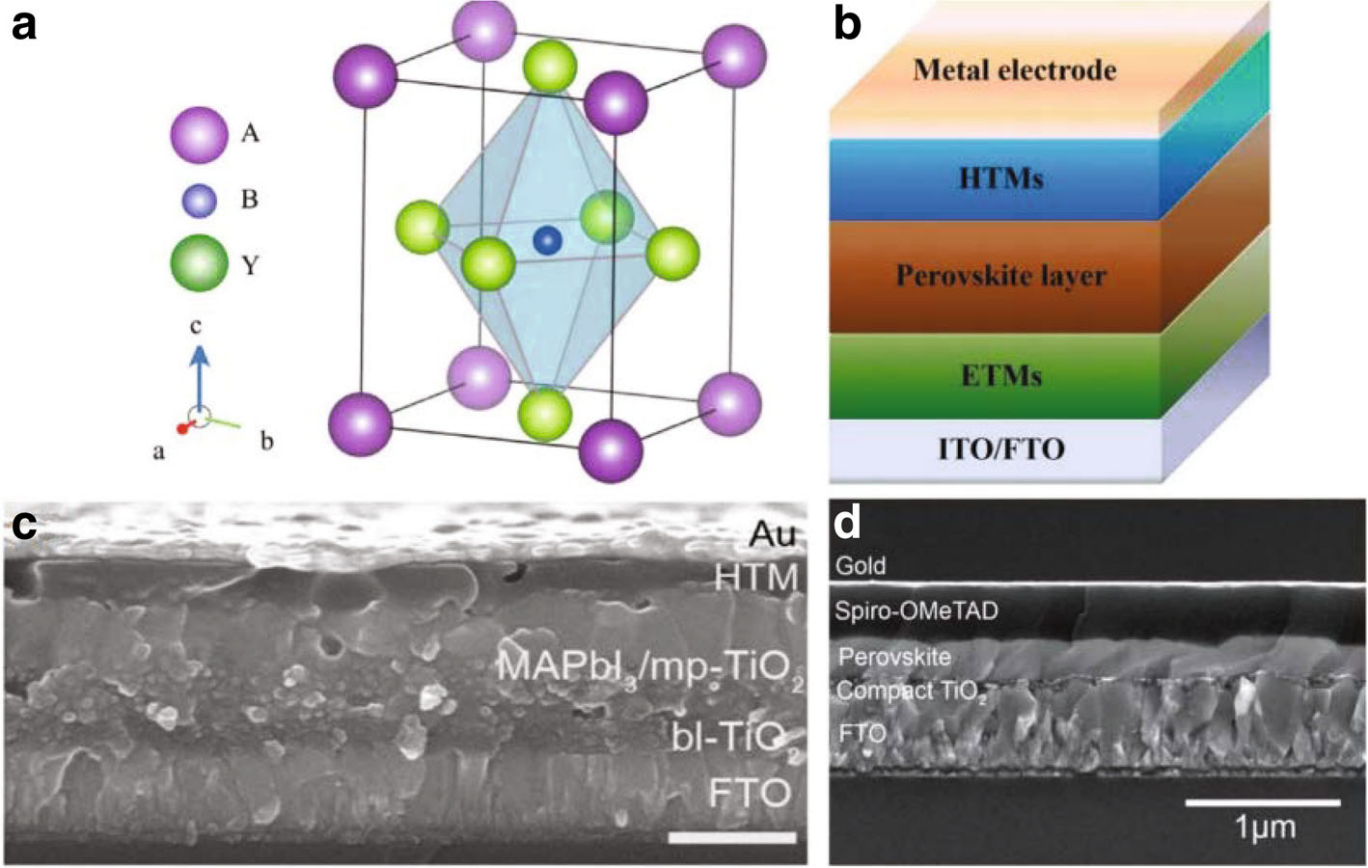
**a** Crystal structure of a perovskite [22]. **b** Schematic diagram of general device [23]. **c** Cross-section scanning electron microscopy (SEM) images of a meso-superstructured perovskite solar cell (*scale bar* is 500 nm) [22]. **d** Cross-section SEM images of a normal planar perovskite solar cells with the presence of an HTL and an ETL [22]

Perovskite originally refers to a kind of ceramic oxides with general molecular formula ABY_3_ discovered by the German mineralogist Gustav Rose in 1839. It was named “perovskite” because it is a calcium titanate(CaTiO_3_) compounds exists in calcium titanium ore [18]. The crystal structure of a perovskite is showed in Fig. 2 a. In 2009, perovskite structured materials were first utilized in solar cells by Miyasaka and his colleagues. They creatively replaced the dye pigment in DSSCs with two organic-inorganic hybrid halide-based perovskites, CH_3_NH_3_PbBr_3_ and CH_3_NH_3_PbI_3_. And, eventually, they gained relatively not considerable power conversion efficiency (PCE) of 3.13 and 3.81%, respectively [13].

However, the work did not gain much attention due to low efficiency and poor stability, which resulted from a hole transport layer (HTL) with liquid electrolyte.

An evolutionary jump then happened in 2012 when Kim, Gratzel and Park et al. [14] used perovskite absorbers as the primary photoactive layer to fabricate solid-state meso-superstructured PSCs. Spiro-MeOTAD and mp-TiO_2_ were used as the hole transport and electron transport materials (HTM/ETM), respectively, in their work and resulted in a relatively high efficiency of 9.7% for the first reported perovskite-based solid-state mesoscopic heterojunction solar cell.

After this breakthrough, the investigation of PSCs became hot gradually in photovoltaic (PV) research in the following years. Eventually, the efficiency of PSCs was promoted to 22.1% in early 2016 [1]. Since the maximum theoretical PCE of the PSCs employing CH_3_NH_3_PbI_3−*x*_Cl_*x*_ is 31.4%, there is still enough space for development [19].

Figure 2 b shows the general configuration of PSCs, which usually comprises a tin-doped indium oxide (ITO)/fluorine-doped tin oxide (FTO) substrate, metal electrode, a perovskite photoactive layer, together with necessary charge transport layers (i.e., a hole transport layer (HTL) [20] and an electron transport layer (ETL) [21]) [22, 23]. Figure 2 c, d shows two main device architectures: meso-superstructured perovskite solar cells (MPSCs) [24], which incorporate a mesoporous layer, and planar perovskite solar cells (PPSCs) in which all layers are planar [25].

The working principle of these PSCs can be briefly summarized in the following ways: perovskite layer absorbs the incident light, generating electron and hole, which are extracted and transported by ETMs and HTMs, respectively. These charge carriers are finally collected by electrodes forming PSCs [23].

## High-Efficiency Perovskite Solar Cells

### Intramolecular Exchange

In June 2015, Woon Seok Yang and his colleagues report an approach for depositing high-quality FAPbI_3_ films with which they fabricated FAPbI_3_ PSCs with a PCE of 20.1% under AM 1.5 G full-sun illuminations [26].

**Fig. 3:**
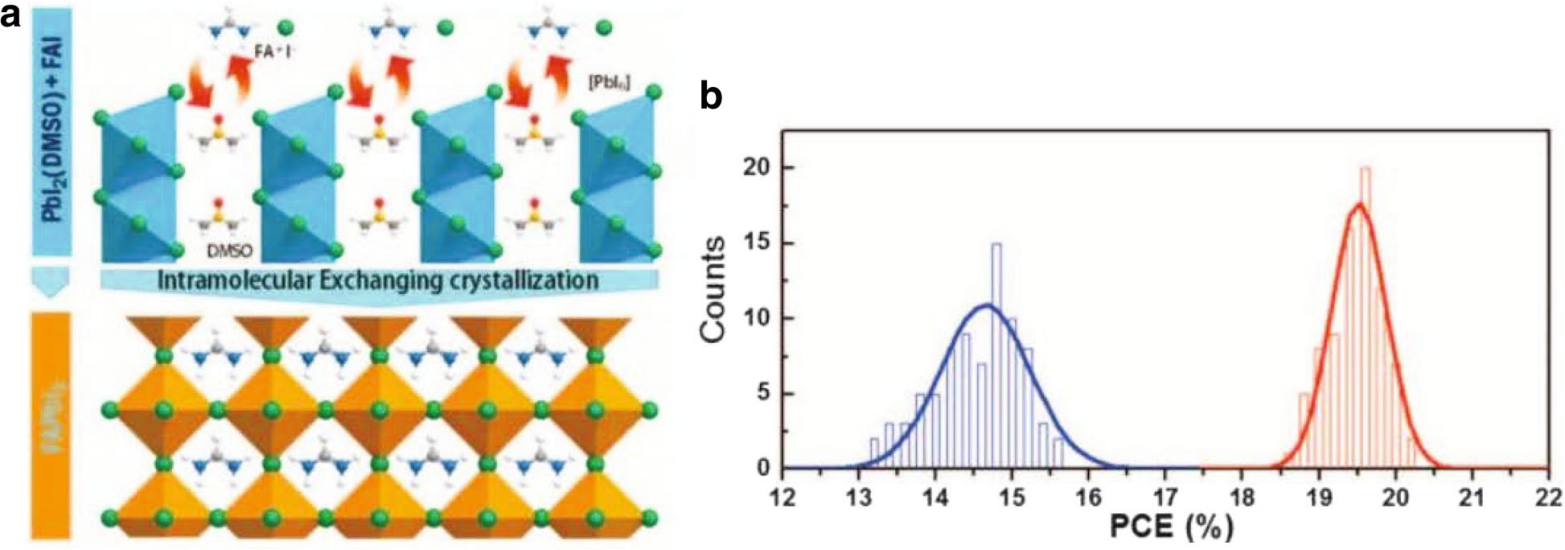
PbI_2_ complex formation and X-ray diffraction. **a** Schematics of FAPbI_3_ perovskite crystallization involving the direct intramolecular exchange of DMSO molecules intercalated in PbI_2_ with formamidinium iodide (FAI). The DMSO molecules are intercalated between edge-sharing [PbI_6_] octahedral layers. **b** Histogram of solar cell efficiencies for each 66 FAPbI_3_-based cells fabricated by IEP and conventional process [26]

On the road to enhance the efficiency of solar cells, the deposition of dense and uniform films is critical for optoelectronic properties of perovskite films and is an important research topic of highly efficient PSCs. Woon Seok Yang and his colleagues report an approach for depositing high-quality FAPbI_3_ films, involving FAPbI_3_ crystallization by the direct intramolecular exchange of dimethyl sulfoxide (DMSO) molecules intercalated in PbI_2_ with formamidinium iodide (Fig. 3). This process produces FAPbI_3_ films with (111)-preferred crystallographic orientation, large-grained dense microstructures, and flat surfaces without residual PbI_2_. Using films prepared by this technique, they fabricated FAPbI_3_-based PSCs with maximum power conversion efficiency greater than 20%.

### Cesium-Containing Triple-Cation Perovskite Solar Cells

Adding inorganic cesium to triple-cation perovskite compositions, Michael Saliba and his colleagues demonstrated a perovskite solar cell which not only possesses higher PCEs of 21.1% but also is more stable, contains less phase impurities, and is less sensitive to processing conditions [27, 28].

**Fig. 4:**
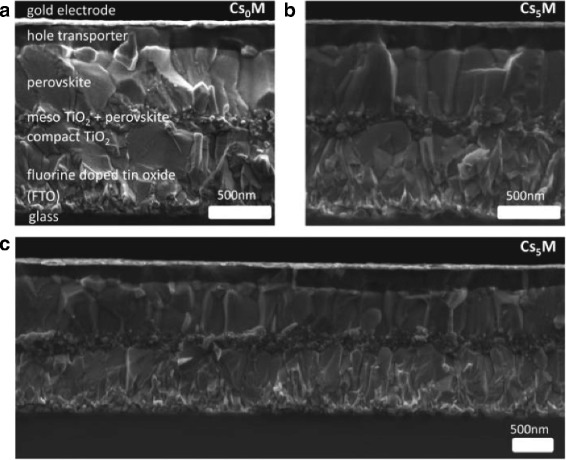
Cross-sectional SEM images of **a** Cs_0_M, **b** Cs_5_M, and **c** low-magnification Cs5M devices [27]

They investigated triple-cation perovskites of the generic form “ Cs_*x*_(MA_0.17_FA_0.83_)_(100−*x*)_Pb(I_0.83_Br_0.17_)_3_,” demonstrating that the use of all three cations, Cs, MA, and FA, provides additional versatility in fine-tuning high-quality perovskite films (Fig. 4). They yielded stabilized PCEs exceeding 21 and 18% after 250 h under operational conditions. Even more, the triple-cation perovskite films are thermally more stable and less affected by fluctuating surrounding variables such as temperature, solvent vapors, or heating protocols. This robustness is important for reproducibility, which is one of the key requirements for cost-efficient large-scale manufacturing of PSCs.

### Graded Bandgap Perovskite Solar Cells

**Fig. 5:**
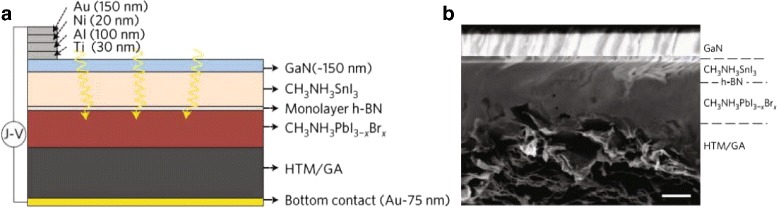
Cross-sectional schematic and SEM images of perovskite cell with integral monolayer h-BN and graphene aerogel. **a** Schematic of a graded bandgap perovskite solar cell. Gallium nitride (GaN), monolayer hexagonal boron nitride (h-BN), and graphene aerogel (GA) are key components of the high-efficiency cell architecture. **b** Cross-sectional SEM image of a representative perovskite device. The division between perovskite layers and the monolayer h-BN is not visible in this SEM image. The *dashed lines* indicate the approximate location of the perovskite layers and the monolayer h-BN as a guide to the eye. The location of perovskite layers and monolayer h-BN is extracted from the related EDAX analysis. Thickness of the CH_3_NH_3_SnI_3_ layer is 150 nm and that of the CH_3_NH_3PbI_3−*x*_Br_*x*_ is 300 nm. *Scale bar*, 200 nm [29]

On November 7, 2016, scientists from University of California, Berkeley, and Lawrence Berkeley National Laboratory reported a new design that already achieved an average steady state efficiency of 18.4%, with a height of 21.7% and a peak efficiency of 26% [29–31]. They use a single-atom thick layer of hexagonal boron nitride to combine two materials into a tandem solar cell and, eventually, obtained high efficiency. The compositions of the perovskite materials are both the organic molecules methyl and ammonia, whereas one contains the metals tin and iodine, while the other contains lead and iodine doped with bromine. The former is tuned to preferentially absorb light with an energy of 1 eV—infrared or heat energy—while the latter absorbs photons of energy 2 eV, or an amber color. Prior to this attempt, the merging of two perovskite materials has failed because the materials degrade one another’s electronic performance. This new way to combine two perovskite solar cell materials into one “graded bandgap” solar cell demonstrated exciting results. The solar cell absorbs nearly the entire spectrum of visible light. This is very beneficial to improve efficiency. The structure is shown in Fig. 5. They found that freshly illuminated cells tend to have higher PCE than cells that have been illuminated for more than a few minutes. For example, for a given graded bandgap perovskite cell, the PCE is between 25 and 26% in the first 2 min of illumination while the cell reaches a “steady state” with a stable PCE of 20.8% after approximately 5 min. This result indicates that perovskite-based solar cells have time-dependent performance characteristics. The measurement of 40 graded bandgap perovskite cells demonstrated that the average steady state PCE over all devices is 18.4% while the best graded bandgap cell in the steady state exhibited a PCE of 21.7%.

## Stability of Perovskite Solar Cells

In recent years, the record efficiency of PSCs has been updated from 9.7 to 22.1%. However, the poor long-term device stability of PSCs is still a big remaining challenge for PSCs, which decide whether exciting achievements could be transferred from the laboratory to industry and outdoor applications. Therefore, long-term stability is an issue that needs to be addressed urgently for PSCs. Quite a number of people have shown interest in the issue of stability and given guiding opinions on improving stability [32–44].

Multiple reports have suggested that moisture and oxygen, UV light, solution processing, and thermal stress are four key factors affecting the stability of PSCs. Observed (sometimes rapid) degradation occurs when devices are exposed to those environmental factors [22, 32, 45, 46].

**Fig. 6:**
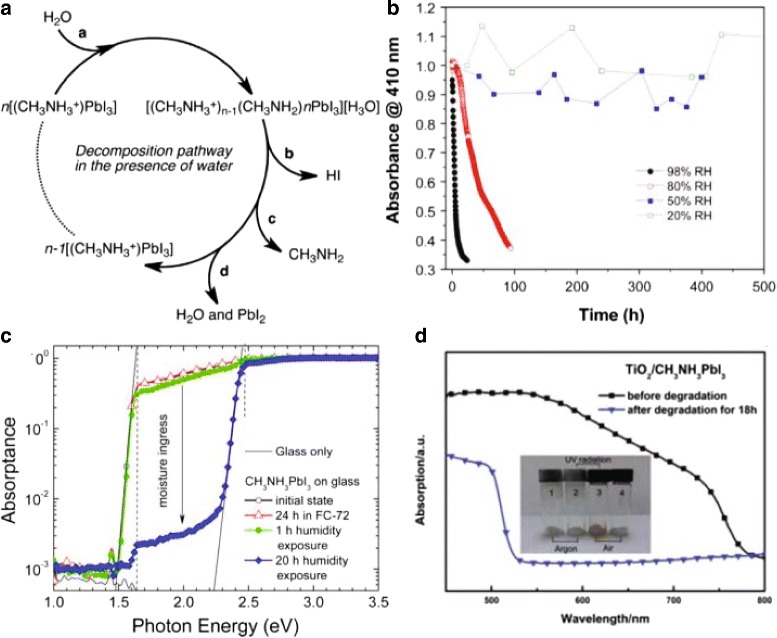
**a** Proposed decomposition pathway of CH_3_NH_3_PbI_3_ in the presence of a water molecule. The main product of this pathway is PbI_2_ [48]. **b** Normalized absorbance measurements (taken at 410 nm) for CH_3_NH_3_PbI_3_ films exposed to different relative humidity [49]. **c** PDS spectra for CH_3_NH_3_PbI_3_ films before (initial state) and after exposure to a relative humidity in the range of 30–40% for different times. This clearly indicates a significant reduction in absorption in the range of 1.5–2.5 eV after exposure to humidity [1]. **d** Degradation of CH_3_NH_3_PbI_3_ in moisture and air atmosphere. UV-vis absorption spectra of TiO_2_/CH_3_NH_3_PbI_3_ film before and after degradation. The *inset* is a photograph of CH3NH3I exposed to different conditions: (1) CH_3_NH_3_I exposed to argon and without UV radiation; (2) CH_3_NH_3_I exposed to argon and with UV radiation; (3) CH_3_NH_3_I exposed to air and with UV radiation; and (4) CH_3_NH_3_I exposed to air and without UV radiation [32]

Guangda Niu and his colleagues [32] expressed their views that in order to modulate the stability of PSCs, many factors should be taken into consideration, including the composition and crystal structure design of the perovskite; the preparation of the HTM layer and electrode materials; the thin film fabrication method, interfacial engineering, and encapsulation methods (multilayer encapsulation or helmet encapsulation); and the module technology. Their work verified that oxygen, together with moisture, could lead to the irreversible degradation of CH_3_NH_3_PbI_3_ which is always employed as sensitizers in PSCs. They expose TiO_2_/CH_3_NH_3_PbI_3_ film to air with a humidity of 60% at 35 °C for 18 h, and then, the absorption between 530 and 800 nm greatly decreased (Fig. 6 d).

Especially, humidity is an indispensable factor when an experimental investigation on the issue of stability is conducted.

Work lead by Kwon et al. shows that the hygroscopic nature of amine salts results from the origin of moisture instability [47]. Figure 6 a shows the likely process of CH_3_NH_3_PbI_3_ decomposition which was displayed by Frost et al. [48]. The process indicates that HI and MA are soluble in water, which directly leads to irreversible degradation of the perovskite layer.

Yang et al. investigated this degradation process by performing in situ absorbance and grazing incidence X-ray diffraction (GIXRD) measurements [49]. To make a valid contrast in degradation, they carefully control the relative humidity (RH) in which the films were measured. Figure 6 b shows their research result of the influence of RH on the film degradation. The absorption reduced to half of its original value in only 4 h for the 98% RH case while this would take 10,000 h extrapolation of the degradation curve for a low RH of 20%. The result indicates expectedly that higher RH values cause a more rapid reduction in film absorption than a low RH. Moreover, further experiment demonstrates that varied carrier gases, N_2_ or air led to no significant change in the degradation of the absorbance, indicating that the main cause of degradation in the perovskite film, under normal atmosphere, is the presence of moisture.

In 2014, De Wolf et al. used another powerful technique, photothermal deflection spectroscopy (PDS), to measure the moisture-induced decomposition of CH_3_NH_3_PbI_3_ [50]. They measured the PDS spectra of CH_3_NH_3_PbI_3_ layers after exposure to ambient air with 30–40% relative humidity during 1 and 20 h, respectively. Figure 6 c shows that the absorptance between photon energies of 1.5 and 2.5 eV drops by two orders of magnitude after exposure to humidity for 20 h. In addition, the absorption edge that occurs at 1.57 eV in its initial state shifts to 2.3 eV, an energy corresponding to the bandgap of PbI_2_ [51], which indicate that CH_3_NH_3_PbI_3_ can decompose into PbI_2_ in a humid ambient due to the dissolution of disordered CH_3_NH_3_I [35, 52].

Many methods are researched for stability enhancement of PSCs recent years. Xin Wang et al. successfully developed a simple solution-processed CeO_*x*_ (x = 1.87) ETL at low temperature. According to their work, CeO_*x*_-based devices exhibit superior stability under light soaking compared to TiO_2_-based PSCs [53]. Zhiping Wang et al. presented the first long-term stability study of the new “mixed-cation mixed-halide” perovskite composition FA_0.83_Cs_0.17_Pb(I_0.6_Br_0.4_)_3_(FA = (HC(NH_2_)_2_)) and discover that the cells are remarkably stable when exposed to full-spectrum simulated sun light in ambient conditions without encapsulation [54]. Han et al. adopted thick carbon as the electrode and the device’s own hole transport layer; the cell was stable for >1000 h in ambient air under full sunlight while it achieved a PCE of 12.8% [55].

## Interface Engineering

The interface is vital to the performance of the devices, since it is not only critical to the exciton formation, dissociation, and recombination but also influences the degradation of devices [56]. As a result, the interface engineering for reduced recombination is extremely important to achieve high-performance and high-stability PSCs.

Tan et al. reported a contact-passivation strategy using chlorine-capped TiO_2_ colloidal nanocrystal film that mitigates interfacial recombination and improves interface binding in low-temperature planar solar cells. The PSCs achieved certified efficiencies of 20.1 and 19.5% for active areas of 0.049 and 1.1 cm^2^, respectively. Moreover, PSCs with efficiency greater than 20% retained 90% of their initial performance after 500 h of continuous room-temperature operation at their maximum power point under 1-sun illumination [57]. Wang and co-workers inserted an insulating tunneling layer between the perovskite and the electron transport layer. The thin insulating layer allowed the transport of photo-generated electrons from perovskite to C_60_ cathode through tunneling and blocked the photo-generated holes back into the perovskite. Devices with these insulating materials exhibited an increased PCE of 20.3% under 1-sun illumination [58]. Correa-Baena et al. provided some theoretical guidance by investigating in depth the recombination at the different interfaces in a PSC, including the charge-selective contacts and the effect of grain boundaries [59].

## Conclusions

The development of PSCs in the last few years makes it a promising alternative for the next-generation, low-cost, and high-efficiency solar cell technology. Driven by the urgent need of cost-effective, high-efficient solar cells, PSCs have been intensively investigated in the recent years. Various kinds of methods are used to improve the performance. We summarize the recent development of high-efficiency PSCs. The recorded efficacy of single-junction PSCs has been increased by a few folds to over 22% in the last few years, approaching the best single crystalline silicon solar cells. Undoubtedly, halide perovskite materials have emerged as an attractive alternative to conventional silicon solar cells. However, the stability issue is still urgent to be solved. The recent progress made in the device architectures and new materials open new opportunities for highly stable PSCs.

## References

[1] Nazeeruddin MK (2016). In retrospect: twenty-five years of low-cost solar cells. Nature.

[2] Green MA (2001). Third generation photovoltaics: ultra-high conversion efficiency at low cost. Prog Photovoltaics Res Appl.

[3] Han H, Huang G, Zhao W, Shi Y (2013). Test and analysis of outdoor power generation performance of crystalline silicon solar cells. Taiyangneng Xuebao/acta Energiae Solaris Sinica.

[4] Song D, Xiong J (2013) Fabrication and characterization of high efficiency bifacial n-type silicon solar cell and pv modules. Acta Energiae Solaris Sinica.

[5] Dore J, Ong D, Varlamov S, Egan R, Green MA (2013). Progress in laser-crystallized thin-film polycrystalline silicon solar cells: intermediate layers, light trapping, and metallization. IEEE J Photovoltaics.

[6] Lu PHD, Lin D, Wang X, Lennon A, Wenham S (2016). Laser doping through anodic aluminium oxide silicon solar cell. Solar Energy Mater Solar Cells.

[7] Kayes BM, Nie H, Twist R, Spruytte SG, Reinhardt F, Kizilyalli IC, Higashi GS (2012) 27.6% conversion efficiency, a new record for single-junction solar cells under 1 sun illumination In: Photovoltaic Specialists Conf, 000004–000008.

[8] Venkatasubramanian R, Timmons ML, Sharps PR, Hutchby JA (1996) 18.2% (am1.5) efficient gaas solar cell on optical-grade polycrystalline ge substrate In: Photovoltaic Specialists Conference, 1993., Conference Record of the Twenty Third IEEE, 691–695.

[9] Keavney CJ, Haven VE, Vernon SM (1990) Emitter structures in mocvd inp solar cells In: Photovoltaic Specialists Conference, 1990., Conference Record of the Twenty First IEEE, 141–1441.

[10] Rauf IA, Rezai P (2017). A review of materials selection for optimized efficiency in quantum dot sensitized solar cells: a simplified approach to reviewing literature data. Renewable Sustainable Energy Rev.

[11] Pan Z, Mora-Seró I, Shen Q, Zhang H, Li Y, Zhao K, Wang J, Zhong X, Bisquert J (2014). High-efficiency “green” quantum dot solar cells. J Am Chem Soc.

[12] Wu J, Yu P, Susha AS, Sablon KA, Chen H, Zhou Z, Li H, Ji H, Niu X, Govorov AO, Rogach AL, Wang ZM (2015). Broadband efficiency enhancement in quantum dot solar cells coupled with multispiked plasmonic nanostars. Nano Energy.

[13] Kojima A, Teshima K, Shirai Y, Miyasaka T (2009). Organometal halide perovskites as visible-light sensitizers for photovoltaic cells. J Am Chem Soc.

[14] Kim HS, Lee CR, Im JH, Lee KB, Moehl T (2012). Lead iodide perovskite sensitized all-solid-state submicron thin film mesoscopic solar cell with efficiency exceeding 9%. Sci Rep.

[15] Jeon NJ, Noh JH, Kim YC, Yang WS, Ryu S, Seok SI (2014). Solvent engineering for high-performance inorganic-organic hybrid perovskite solar cells. Nat Mater.

[16] Green MA, Ho-Baillie A, Snaith HJ (2014). The emergence of perovskite solar cells. Nat Photon.

[17] O’Regan B, Gratzel M (1991). A low-cost, high-efficiency solar cell based on dye-sensitized colloidal TiO_2_ films. Nature.

[18] Rose G (1839) De Novis Quibusdam Fossilibus Quae in Montibus Uraliis Inveniuntur Scripsit.

[19] Yin WJ, Yang JH, Kang J, Yan Y, Wei SH (2015). Halide perovskite materials for solar cells: a theoretical review. J Mater Chem A.

[20] Swetha T, Singh SP (2015). Perovskite solar cells based on small molecule hole transporting materials. J Mater Chem A.

[21] Liu H, Huang Z, Wei S, Zheng L, Xiao L, Gong Q (2016). Nano-structured electron transporting materials for perovskite solar cells. Nanoscale.

[22] Yang L, Barrows AT, Lidzey DG, Wang T (2016). Recent progress and challenges of organometal halide perovskite solar cells. Rep Progress Phys.

[23] Tong X, Lin F, Wu J, Wang ZM (2015). High performance perovskite solar cells. Adv Sci.

[24] Jeon NJ, Lee J, Noh JH, Nazeeruddin MK, Gratzel M, Seok SI (2013). Efficient inorganic–organic hybrid perovskite solar cells based on pyrene arylamine derivatives as hole-transporting materials. J Am Chem Soc.

[25] Eperon GE, Stranks SD, Menelaou C, Johnston MB, Herz LM, Snaith HJ (2014). Formamidinium lead trihalide: a broadly tunable perovskite for efficient planar heterojunction solar cells. Energy Environ Sci.

[26] Yang WS, Noh JH, Jeon NJ, Kim YC, Ryu S, Seo J, Seok SI (2015). High-performance photovoltaic perovskite layers fabricated through intramolecular exchange. Science.

[27] Saliba M, Matsui T, Seo JY, Domanski K, Correa-Baena JP, Nazeeruddin MK, Zakeeruddin SM, Tress W, Abate A, Hagfeldt A (2016). Cesium-containing triple cation perovskite solar cells: improved stability, reproducibility and high efficiency. Energy Environ Sci.

[28] Breakthrough Reproducibility Achieved for Perovskite Solar Cells. https://www.perovskite-info.com/breakthrough-reproducibility-achieved-perovskite-solar-cells. Accessed 2016.

[29] Ergen O, Gilbert SM, Pham T, Turner SJ, Tan MTZ, Worsley MA, Zettl A (2017). Graded bandgap perovskite solar cells. Nat Mater.

[30] Flexible perovskite-perovskite solar cells reach 21.7% efficiency. https://www.perovskite-info.com/flexible-perovskite-perovskite-solar-cells-reach-217-efficiency. Accessed 2016.

[31] Major advance in solar cells made from cheap, easy-to-use perovskite. http://news.berkeley.edu/2016/11/07/major-advance-in-solar-cells-made-of-cheap-easy-to-use-perovskite/. Accessed 2016.

[32] Niu G, Guo X, Wang L (2015). Review of recent progress in chemical stability of perovskite solar cells. J Mater Chem A.

[33] Hailegnaw B, Kirmayer S, Edri E, Hodes G, Cahen D (2015). Rain on methylammonium lead iodide based perovskites: possible environmental effects of perovskite solar cells. J Phys Chem Lett.

[34] Leijtens T, Eperon GE, Pathak S, Abate A, Lee MM, Snaith HJ (2013). Overcoming ultraviolet light instability of sensitized TiO_2_ with meso-superstructured organometal tri-halide perovskite solar cells. Nat Commun.

[35] Noh JH, Im SH, Heo JH, Mandal TN, Seok SI (2013). Chemical management for colorful, efficient, and stable inorganic–organic hybrid nanostructured solar cells. Nano Lett.

[36] Law C, Miseikis L, Dimitrov S, Shakya-Tuladhar P, Li X, Barnes PR, Durrant J, O’Regan BC (2014). Performance and stability of lead perovskite/ TiO_2_, polymer/PCBM, and dye sensitized solar cells at light intensities up to 70 suns. Adv Mater.

[37] Li X, Dar MI, Yi C, Luo J, Tschumi M, Zakeeruddin SM, Nazeeruddin MK, Han H, Grätzel M (2015). Improved performance and stability of perovskite solar cells by crystal crosslinking with alkylphosphonic acid *ω*-ammonium chlorides. Nat Chem.

[38] Kaltenbrunner M, Adam G, Głowacki ED, Drack M, Schwödiauer R, Leonat L, Apaydin DH, Groiss H, Scharber MC, White MS (2015). Flexible high power-per-weight perovskite solar cells with chromium oxide-metal contacts for improved stability in air. Nat Mater.

[39] Smith IC, Hoke ET, Solis-Ibarra D, McGehee MD, Karunadasa HI (2014). A layered hybrid perovskite solar-cell absorber with enhanced moisture stability. Angewandte Chemie.

[40] Wu CG, Chiang CH, Tseng ZL, Nazeeruddin MK, Hagfeldt A, Grätzel M (2015). High efficiency stable inverted perovskite solar cells without current hysteresis. Energy Environ Sci.

[41] Aharon S, Gamliel S, El Cohen B, Etgar L (2014). Depletion region effect of highly efficient hole conductor free CH_3_NH_3_PbI_3_ perovskite solar cells. Phys Chem Chem Phys.

[42] Chen W, Wu Y, Yue Y, Liu J, Zhang W, Yang X, Chen H, Bi E, Ashraful I, Grätzel M (2015). Efficient and stable large-area perovskite solar cells with inorganic charge extraction layers. Science.

[43] Cao J, Yin J, Yuan S, Zhao Y, Li J, Zheng N (2015). Thiols as interfacial modifiers to enhance the performance and stability of perovskite solar cells. Nanoscale.

[44] Jiang Q, Rebollar D, Gong J, Piacentino EL, Zheng C, Xu T (2015). Pseudohalide-induced moisture tolerance in perovskite CH_3_NH_3_Pb(SCN)_2_I thin films. Angewandte Chemie.

[45] Leijtens T, Eperon GE, Noel NK, Habisreutinger SN, Petrozza A, Snaith HJ (2015). Stability of metal halide perovskite solar cells. Adv Energy Mater.

[46] Rong Y, Liu L, Mei A, Li X, Han H (2015). Beyond efficiency: the challenge of stability in mesoscopic perovskite solar cells. Adv Energy Mater.

[47] Kwon YS, Lim J, Yun HJ, Kim YH, Park T (2014). A diketopyrrolopyrrole- containing hole transporting conjugated polymer for use in efficient stable organic–inorganic hybrid solar cells based on a perovskite. Energy Environ Sci.

[48] Frost JM, Butler KT, Brivio F, Hendon CH, Van Schilfgaarde M, Walsh A (2014). Atomistic origins of high-performance in hybrid halide perovskite solar cells. Nano Lett.

[49] Yang J, Siempelkamp BD, Liu D, Kelly TL (2015). Investigation of CH_3_NH_3_PbI_3_ degradation rates and mechanisms in controlled humidity environments using in situ techniques. ACS Nano.

[50] De Wolf S, Holovsky J, Moon SJ, Loper P, Niesen B, Ledinsky M, Haug FJ, Yum JH, Ballif C (2014). Organometallic halide perovskites: sharp optical absorption edge and its relation to photovoltaic performance. J Phys Chem Lett.

[51] Liu D, Kelly TL (2014). Perovskite solar cells with a planar heterojunction structure prepared using room-temperature solution processing techniques. Nat Photonics.

[52] Kim HS, Im SH, Park NG (2014). Organolead halide perovskite: new horizons in solar cell research. J Phys Chem C.

[53] Wang X, Deng LL, Wang LY, Dai SM, Xing Z, Zhan XX, Lu XZ, Xie SY, Huang RB, Zheng LS (2017). Cerium oxide standing out as an electron transport layer for efficient and stable perovskite solar cells processed at low temperature. J Mater Chem A.

[54] Wang Z, McMeekin DP, Sakai N, van Reenen S, Wojciechowski K, Patel JB, Johnston MB, Snaith HJ (2017) Efficient and air-stable mixed-cation lead mixed-halide perovskite solar cells with n-doped organic electron extraction layers. Adv Mater 29(5): 1604186. doi:10.1002/adma.201604186. 1604186 10.1002/adma.201604186 27905138

[55] Mei A, Li X, Liu L, Ku Z, Liu T, Rong Y, Xu M, Hu M, Chen J, Yang Y, Grätzel M, Han H (2014). A hole-conductor–free, fully printable mesoscopic perovskite solar cell with high stability. Science.

[56] Niu G, Li W, Li J, Wang L (2016). Progress of interface engineering in perovskite solar cells. Sci China Mater.

[57] Tan H, Jain A, Voznyy O, Lan X, García de Arquer FP, Fan JZ, Quintero-Bermudez R, Yuan M, Zhang B, Zhao Y, Fan F, Li P, Quan LN, Zhao Y, Lu ZH, Yang Z, Hoogland S, Sargent EH (2017). Efficient and stable solution-processed planar perovskite solar cells via contact passivation. Science.

[58] Wang Q, Dong Q, Li T, Gruverman A, Huang J (2016). Thin insulating tunneling contacts for efficient and water-resistant perovskite solar cells. Adv Mater.

[59] Correa-Baena JP, Tress W, Domanski K, Anaraki EH, Turren-Cruz SH, Roose B, Boix PP, Gratzel M, Saliba M, Abate A, Hagfeldt A (2017). Identifying and suppressing interfacial recombination to achieve high open-circuit voltage in perovskite solar cells. Energy Environ Sci.

[60] Agency IE (2015) Energy and climate change: world energy outlook special report. Int Energy Agency.

